# Digital Chromoendoscopy for Diagnosis of Diminutive Colorectal Lesions

**DOI:** 10.1155/2012/279521

**Published:** 2012-10-03

**Authors:** Carlos Eduardo Oliveira dos Santos, Daniele Malaman, César Vivian Lopes, Júlio Carlos Pereira-Lima, Artur Adolfo Parada

**Affiliations:** ^1^Department of Endoscopy and Gastroenterology, Dr Mario Araujo University Hospital, 96400-130 Bagé, RS, Brazil; ^2^Department of Endoscopy and Gastroenterology, Santa Casa Hospital and Fundação Riograndense Universitária de Gastroenterologia (FUGAST), 90610-210 Porto Alegre, RS, Brazil; ^3^Department of Endoscopy 9 de Julho Hospital, 01409-902 São Paulo, SP, Brazil

## Abstract

*Introduction*. To compare the accuracy of digital and real-time chromoendoscopy for the differential diagnosis of diminutive (<5 mm) neoplastic and nonneoplastic colorectal lesions. *Materials and Methods*. This is a prospective randomized study comparing the Fujinon intelligent color enhancement (FICE) system (65 patients/95 lesions) and indigo carmine (69 patients/120 lesions) in the analysis of capillary meshwork and pit pattern, respectively. All lesions were less than 5 mm in diameter, and magnification was used in both groups. Histopathology was the gold standard examination. *Results*. Of 215 colorectal lesions, 153 (71.2%) were adenomas, and 62 were hyperplastic polyps (28.8%). Morphological analysis revealed 132 (61.4%) superficial lesions, with 7 (3.3%) depressed lesions, and 83 (38.6%) protruding lesions. Vascular meshwork analysis using FICE and magnification resulted in 91.7% sensitivity, 95.7% specificity, and 92.6% accuracy in differentiating neoplastic from nonneoplastic lesions. Pit pattern analysis with indigo carmine and magnification showed 96.5% sensitivity, 88.2% specificity, and 94.2% accuracy for the same purpose. *Conclusion*. Both magnifying virtual chromoendoscopy and indigo carmine chromoendoscopy showed high accuracy in the histopathological diagnosis of colorectal lesions less than 5 mm in diameter.

## 1. Introduction

Colorectal cancer is one of the most commonly diagnosed malignancies in Western countries and represents a major cause of morbidity and mortality associated with cancer [1]. The prevention of colorectal cancer requires the diagnosis and resection of precursor lesions, according to the adenoma-carcinoma sequence [2]. It is also important to consider the pathway of the *de novo* cancer (carcinoma without prior adenomatous tissue) because small (even diminutive) lesions, especially depressed ones, may be more malignant, even showing invasive behavior [3]. A diagnosis of early cancer is possible only if the minimal changes in structure and color (pale color or hyperemia) displayed on the mucosal surface of the lesion can be recognized [4]. Endoscopic detection and treatment of these neoplasms is the most cost-effective strategy to reduce the incidence and mortality of colorectal cancer [5, 6]. Colonoscopy is the best diagnostic method, and the use of chromoendoscopy (CE) with indigo carmine and crystal violet may help characterize the morphology of lesions, whose correct interpretation is crucial in choosing the appropriate treatment. When associated with magnification, this method can provide high accuracy in differentiating neoplastic from non-neoplastic lesions after pit pattern analysis [7–12], in the assessment of invasion depth of carcinomas [13], and in the diagnosis of diminutive residual tumor after endoscopic resection [14], thus increasing the efficiency of the endoscopic procedure [15]. Some studies indicate that CE may increase the detection rate of small flat adenomas in diagnostic colonoscopies [16, 17]. 

In recent years, new technologies have emerged and enabled the analysis of surface (pit-like pattern) and microvascular patterns, at the push of a button on the colonoscope and without the need for dyes, to achieve excellent results in the differential diagnosis between nonneoplastic and neoplastic colorectal lesions [18, 19] and to determine the depth of invasion of early cancer [20, 21]. This technology is known as digital chromoendoscopy (DCE) and, similarly to CE, allows a predictive *in vivo* histological diagnosis, reducing time and effort. New DCE techniques include the Fujinon intelligent color enhancement (FICE) system, narrow-band imaging (NBI), Olympus, and, more recently, i-Scan developed by Pentax. FICE and i-Scan systems are based on a computed spectral estimation technology that processes the reflected photons to reconstitute virtual images for a choice of different wavelengths of red, green, and blue signaling. The NBI system depends on optical filters within the light source, using a frame sequential lighting method.

Several series using this technology have shown similar results for CE and DCE, especially when associated with magnification [22–24], and some findings have demonstrated increased detection of small nonpolypoid neoplastic lesions using DCE when compared with conventional colonoscopy [25–27]. This study aimed to evaluate the ability of DCE (using the FICE system) to differentiate between neoplastic and non-neoplastic lesions less than 5 mm in diameter and to compare FICE accuracy with that of real-time CE (using indigo carmine) in the investigation of colorectal lesions.

## 2. Materials and Methods

Between December 2007 and June 2008, this prospective randomized study analyzed 215 colorectal lesions less than 5 mm in 134 patients (74 women; mean age, 60.6 years).

Patients were eligible for study participation if they were indicated for colon cancer screening or had diagnostic colonoscopy for abdominal pain. Exclusion criteria were coagulopathy, incomplete colonoscopy, poor bowel preparation, polyps with 5 mm or more, history or presence of inflammatory bowel disease, polyposis syndrome, and rectal bleeding in the last 6 months, patients with previous colonoscopy or surgical resection of colon or rectum.

Patients were randomly allocated by sealed envelope to colonoscopy with targeted magnification FICE (group I) or with indigo carmine chromoendoscopy (group II).

All lesions were analyzed with a high-resolution magnifying colonoscope (Fujinon 490ZW5, Fujinon Corp., Saitama, Japan), approximately 80–100X, equipped with the EPX 4400 processor. Only after examination under white light, the lesions were analyzed by DCE or real-time CE. Tap water was used to clean the surface when necessary to ensure optimal image quality.

**Figure 1:**
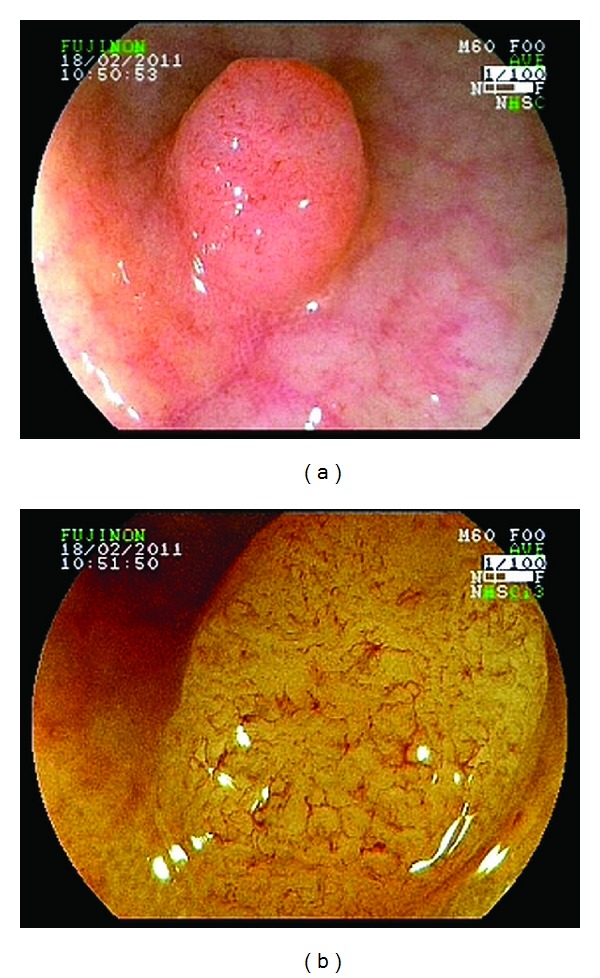
(a) Standard endoscopic image of colorectal lesion type 0-Is. (b) FICE-magnifying observation image of the same lesion: a fine and regular meshwork of brown vessels (positive vascular pattern). Histopathology diagnosed a tubular adenoma.

In group I, 95 lesions in 65 patients were assessed with the FICE system at red, green, and blue wavelengths of R550(2), G500(3), and B470(2), respectively, used to evaluate the capillary pattern, which was defined as either negative (pale-colored surface and invisible vessels or only minute, thin, superficial capillaries) or positive (darkening of the mucosal pattern or a fine meshwork of brown/bluish vessels). Negative capillary meshwork is considered the typical pattern for non-neoplastic lesions and positive capillary meshwork for neoplastic lesions (Figure 1).

**Figure 2:**
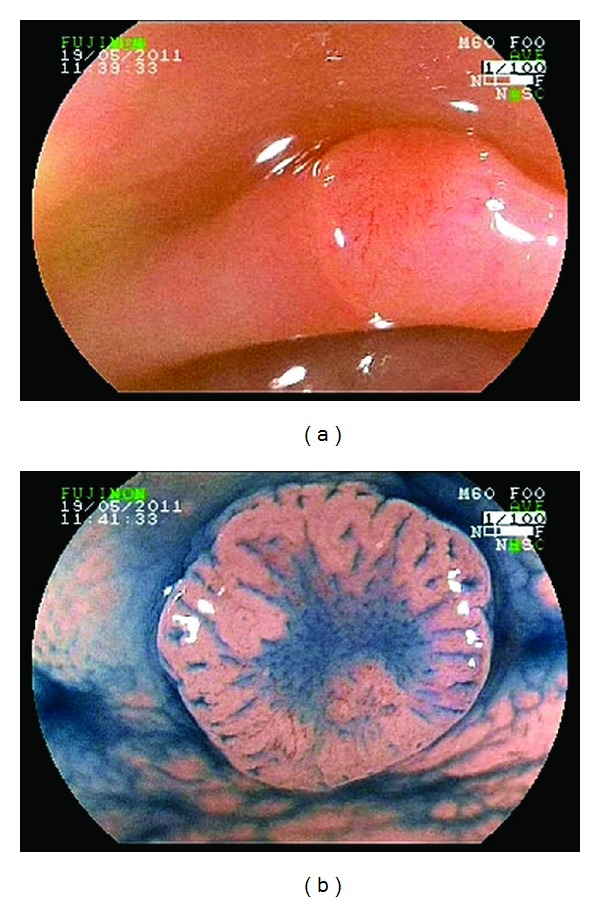
(a) White light image of flat lesion. (b) Indigo carmine dye spraying magnifying showed a lesion type 0-IIa + IIc and the presence of type III L+ IIIs pit pattern. Histopathology identified a tubular adenoma.

In group II, 120 lesions in 69 patients were examined under magnification after 0.8% indigo carmine with a dye spray catheter. The pit pattern analysis was based on the Kudo classification [28, 29], with type I or II pit pattern defining non-neoplastic and type III-V pit pattern defining neoplastic lesions (Figure 2).

All the procedures were performed by a single endoscopist (CEOS) who has routinely used magnifying colonoscopy for over 10 years. The transparent cap was not used. 

Bowel preparation consisted of one-day clear liquid diet, with 10% mannitol solution, being considered appropriate in all patients. Procedures were performed with the patient under conscious sedation (intravenous midazolam and meperidine or fentanyl). 

The morphology of lesions was determined according to the Paris classification [30]. Lesion size was estimated by comparison with the span of open (7 mm) biopsy forceps (FB-24U-1; Olympus Medical Systems Corp., Tokyo, Japan). The location was estimated by the anatomic landmarks.

All lesions were removed with biopsy forceps or by endoscopic mucosal resection, and specimens were fixed in 10% formalin and histologically examined using hematoxylin and eosin staining. Histologic diagnosis was performed by a pathologist blinded to colonoscopic results, and his definitions followed the guidelines of the World Health Organization classification of colorectal tumors [31]. In the case of multiple lesions in the same patient, each lesion was identified individually and placed in different flasks.

The study was performed in accordance with the principles of the Declaration of Helsinki, and written informed consent was obtained from all patients before endoscopy.

### 2.1. Statistical Analysis

For real-time CE with indigo carmine dye spraying and DCE using the FICE system, sensitivity, specificity, positive (PPV), and negative predictive values (NPV), with their 95% confidence intervals (CI), for the diagnosis of colorectal lesions were analyzed by comparing endoscopic diagnoses with histopathology findings (80% power; 5% significance level). Numerical variables were expressed as means and categorical variables as percentage. The Mann-Whitney *U* test was used for comparison of means. Fisher's exact test or the *χ* ^2^ test was used to compare prevalence of neoplasms according to the different imaging methods. A *P* value of less than 0.05 (*P* < 0.05) was considered to be statistically significant. Data were analyzed using the Stata 9.2 Statistical Package.

## 3. Results

**Figure 3:**
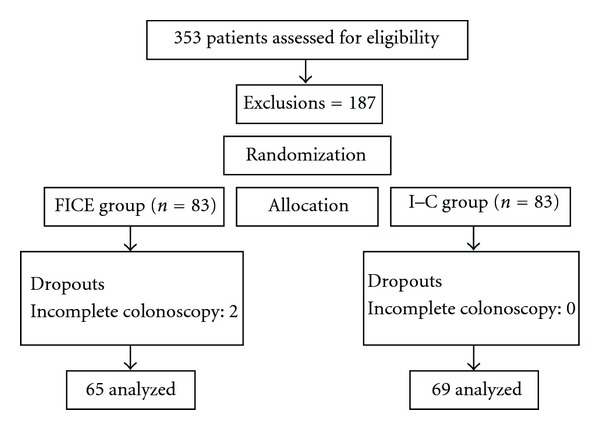
Study group.

From 353 consecutive patients, 166 met primary study criteria. Thirty-two other cases were excluded after randomization due to failure to reach the cecum (*n* = 2), poor bowel preparation (at least semisolid stools at colonoscopy) (*n* = 5), or presence of lesions greater than 5 mm (*n* = 25). Finally, 134 patients with 215 colorectal lesions were analyzed (Figure 3). 

Of 215 colorectal lesions, 153 (71.2%) were neoplastic lesions; of these, 125 (58.1%) were tubular adenomas. The mean size of all adenomas was 3.2 mm (DP = 1.1). All non-neoplastic lesions (62/28.8%) were hyperplastic polyps. The mean size of misdiagnosed lesions was 2.6 mm in diameter.

Regarding morphology, lesions were classified as follows: 132 (61.4%) superficial lesions, 116 (53.9%) type 0-IIa, 9 (4.2%) 0-IIa + dep, 4 (1.9%) 0-IIa + IIc, 2 (0.9%) 0-IIc + IIa, and 1 (0.5%) 0-IIc; and 83 (38.6%) protruding lesions, 78 (36.3%) 0-Is, 3 (1.4%) 0-Isp, and 2 (0.9%) 0-Ip.

Regarding lesion site, 39 (18.1%) lesions were located in the rectum, 48 (22.3%) in the sigmoid colon, 40 (18.6%) in the descending colon, 36 (16.7%) in the transverse colon, 38 (17.7%) in the ascending colon, and 14 (6.5%) in the cecum.

The characteristics of patients and lesions analyzed are shown in Table 1. There were no significant differences between groups regarding sex, age, or lesion site, morphology, and histology.

In group I (FICE), mean age was 60.2 years, 35 (53.8%) patients were female, and 30 (46.2%) were male. There were 67 (70.5%) neoplastic lesions—57 (60%) tubular adenomas, 9 (9.5%) tubulovillous adenomas, and 1 (1.1%) serrated adenoma—and 28 (29.5%) hyperplastic lesions. Of all 95 lesions, 55 (57.9%) were superficial, and 40 (42.1%) were protruding lesions. Nineteen (20%) lesions were located in the rectum, 41 (43.2%) in the left colon (sigmoid and descending), and 35 (36.8%) in the right colon (transverse, descending, and cecum). In the capillary pattern analysis, 67/95 lesions were classified as positive, 66 histologically confirmed as neoplastic lesions, and 28/95 were classified as negative, 22 confirmed as non-neoplastic lesions (Table 2). DCE with the FICE system showed 91.7% sensitivity, 95.7% specificity, 92.6% accuracy, 98.5% PPV, and 78.6% NPV in differentiating neoplastic from nonneoplastic lesions.

In group II (indigo carmine), mean age was 60.9 years, 39 (56.5%) patients were female, and 30 (43.5%) were male. Of 120 lesions, 86 (71.7%) were neoplastic lesions, of which 68 (56.7%) were tubular adenomas. Seventy-seven (64.2%) were superficial lesions. Twenty (16.7%) lesions were located in the rectum, 47 (39.2%) in the left colon, and 53 (44.2%) in the right colon. Real-time CE using indigo carmine dye spraying to evaluate pit pattern showed 96.5% sensitivity, 88.2% specificity, 94.2% accuracy, 95.4% PPV, and 90.9% NPV in differentiating neoplastic from non-neoplastic lesions.

Reproducibility and validity coefficients for the diagnosis of neoplastic lesions are described in Table 3.

The accuracy was 91% and 94% for capillary meshwork and pit pattern analysis in the diagnosed adenomas of the colon and rectum, respectively.

When comparing accuracy between superficial lesions and protruding lesions, the results are shown in Table 4.

## 4. Discussion

The key to reducing the incidence of colorectal carcinoma is early detection of cancers of the colon and rectum, allowing the endoscopic treatment of these tumors. A representative part of small lesions is hyperplastic polyps, which have no malignant potential and therefore do not require resection. The ability to differentiate neoplastic from non-neoplastic lesions contributes to avoid unnecessary resections and to reduce costs and test time, as well as procedure-related complications. Therefore, ideally, endoscopic resection should be indicated only in neoplastic lesions.

CE with indigo carmine and magnification is used to evaluate pit patterns and has shown good results in discriminating between neoplastic and non-neoplastic lesions, with accuracy ranging from 84 to 96.8%, sensitivity from 91.4–97.6%, and specificity from 67.2–93.9% [9–12]. However, real-time CE is a relatively laborious, time-consuming process, and its learning curve for interpreting pit patterns is considered slow. Thus, most endoscopists do not use CE because they consider it a laborious technique which can disrupt the flow of their routine examinations.

DCE has been recently developed, allowing more detailed examination of the mucosal surface and small superficial capillaries. FICE, i-Scan and NBI are noninvasive technologies that allow a faster, easier, and simpler analysis than CE. At the push of a button on the endoscope, it is possible to assess capillary and surface patterns of colorectal lesions. DCE has yielded overall satisfactory results, similar to those obtained with CE [22–24].

Experience with virtual or indigo carmine chromoendoscopy and colonoscopy without magnification presented a diagnostic accuracy between 68% and 93% [32–35]; however, most studies in the literature using magnification revealed better results.

 A multicenter, prospective, randomized study reported that DCE (NBI) was able to detect more subjects with adenomas (*P* = 0.014), flat adenomas (*P* = 0.003), and right-sided adenomas (*P* = 0.003) compared with white-light colonoscopy. There was no difference in the total number of subjects with advanced adenomas (≥1.0 cm, villous, and high-grade dysplasia) (*P* = 0.216). NBI had longer withdrawal time (0.003), which may have contributed to these better results. Accuracy, sensitivity, and specificity for adenomas ≤5 mm were 79.4%, 85.9%, and 72.2%, respectively [25]. A meta-analysis by Jin et al. [26] also showed that endoscopy with the NBI system significantly increased the rate of flat adenoma detection compared with conventional colonoscopy, and withdrawal time was longer (*P* = 0.0006). Inoue et al. [27] compared conventional colonoscopy and pan-colonic NBI and showed that the latter significantly increased the number of adenomas detected (*P* < 0.05) and the number of diminutive (<5 mm) adenomas detected (*P* < 0.05).

The vessels of the microvascular structure of the normal colorectal epithelium are from 5 to 10 *μ*m in diameter. Magnification may facilitate the recognition of minute surface capillaries, favoring the differential diagnosis between neoplastic and non-neoplastic lesions of the colon and rectum. However, visualization of the capillary pattern of lesions less than 5 mm in diameter is not easy, which may justify our mistaken judgments in the endoscopic evaluation of the capillary meshwork. The mean size of misdiagnosed lesions in our study was 2.6 mm.

Pohl et al. [36] compared the FICE system with low and high magnifications in the identification of adenomas and revealed a greater sensitivity, specificity, and accuracy using high magnification. The results were comparable to those using indigo carmine and higher than those using standard magnification. Kim et al. [37] also observed a significantly greater accuracy with high magnification and FICE for both small and diminutive lesions (*P* < 0.05). These findings are in accordance with a large number of articles showing higher diagnostic accuracy with high versus low magnifications [8–10, 24].

In our previous prospective randomized series on small lesions, using magnification and comparing DCE with CE, accuracy was similar for both methods: 92.8% (capillary pattern) and 90.1% (pit pattern) with the FICE system, and 94.9% with indigo carmine [38]. Likewise, the study by Su et al. [22] showed the same values for sensitivity, specificity, and accuracy (95.7%, 87.5%, and 92.7%, resp.) for both DCE and real-time CE. In our study of lesions less than 5 mm, DCE with high magnification showed 92.6% accuracy, 91.7% sensitivity, and 95.7% specificity, being as effective as real-time CE with indigo carmine in the analysis of the capillary meshwork (Table 3).

This study used a simplified classification based on two groups (positive or negative capillary meshwork), which has also been used by other authors with an accuracy above 90% [19, 34, 39]. As in our previous studies [12, 38], we performed DCE or real-time CE only after detection of lesions under high-definition white-light examination.

The Kudo classification was not designed to be used with DCE though some papers [12, 38, 40] have already shown that it could be employed for evaluation of the pit pattern, as well as the the capillary pattern with good results. East et al. [40] detected a very good agreement between both methods (kappa = 0.83), and, besides, the combination of both methods for evaluation of the pit and capillary patterns increased the sensitivity (*P* = 0.06) once compared to the single analysis of the pit pattern, although with a minimal difference (*P* = 0.50) when compared to the analysis of the capillary pattern. In our study, we did not evaluate the combination of both methods, but we do not believe that they could improve significantly the diagnostic accuracy of the DCE. Limitations of this study are as follow: no intraobserver evaluation, and the procedures were performed by a single endoscopist experienced in colonoscopy, especially DCE, whose experience may have influenced the results.

Yoshida et al. [34] studied the surface pattern of 151 polyps using the FICE system without magnification and reported an accuracy of 89.4% for lesions < 10 mm, and, when assessing lesions < 5 mm, accuracy was 82.7%. Teixeira et al. [41], using their classification of capillary pattern and FICE with magnification in 309 colorectal lesions, found a high accuracy of 98.3%, sensitivity of 99.2%, and specificity of 94.9%. Meta-analyses have shown high diagnostic accuracy using DCE in colorectal lesions in the assessment of both pit and capillary patterns, with no differences between them [42, 43], as demonstrated in our pilot study, in which we reported an accuracy of 94.9% and 93.6%, respectively [12]. Studies using i-Scan have shown accuracy, sensitivity, and specificity ranging from 86.1 to 98.6%, 87.7 to 98% and 84.1 to 100% in differentiating neoplastic from non-neoplastic lesions [23, 44, 45]. In a prospective series comparing NBI versus i-Scan in the histological prediction of diminutive adenomas, no significant differences were evident between the two technologies (accuracy, 87.8% versus 90.7%; sensitivity, 88.8% versus 94.6%; specificity, 86.8% versus 86.4%), but both showed a difference in relation to high-definition white-light colonoscopy (*P* = 0.046 and *P* = 0.017, resp.) [46].

To date, some classifications have been proposed for the assessment of the capillary pattern [20, 39, 41], and even for surface patterns [34], but a reference classification, such as the Kudo classification [28, 29] for pit pattern analysis, has yet to be established.

In conclusion, technological advances are tools that help characterize and differentiate lesions of the colon and rectum, playing a role in the choice of appropriate treatment and avoiding unnecessary procedures. DCE and indigo carmine CE, both associated with magnification, showed high accuracy in the histopathological diagnosis of colorectal lesions less than 5 mm in diameter.

## Figures and Tables

**Table 1:** Characteristics of colorectal lesions analyzed by digital chromoendoscopy (FICE group) and real-time chromoendoscopy (indigo carmine group).

Variable	FICE (95) *n* (%)	Indigo carmine (120) *n* (%)
Sex		
Female	35 (53.8)	39 (56.5)
Male	30 (46.2)	30 (43.5)
Mean age (years)	60.2	60.9
Histopathology		
Neoplastic	67 (70.5)	86 (71.7)
Nonneoplastic	28 (29.5)	34 (28.3)
Histopathology		
Tubular adenoma	57 (60)	68 (56.7)
Tubulovillous adenoma	9 (9.5)	14 (11.6)
Serrated adenoma	1 (1.1)	4 (3.3)
Hyperplastic polyp	28 (29.5)	34 (28.3)
Macroscopic type		
Flat	55 (57.9)	77 (64.2)
Protruded	40 (42.1)	43 (35.8)
Macroscopic classification		
0-IIa	44 (46.3)	72 (60)
0-IIa + dep	7 (7.4)	2 (1.7)
0-IIa + IIc	2 (2.1)	2 (1.7)
0-IIc + IIa	1 (1.1)	1 (0.8)
0-IIc	1 (1.1)	0
0-Is	37 (38.9)	41 (34.1)
0-Isp	1 (1.1)	2 (1.7)
0-Ip	2 (2.1)	0
Location		
Rectum	19 (20)	20 (16.7)
Sigmoid colon	23 (24.2)	25 (20.8)
Descending colon	18 (18.9)	22 (18.3)
Transverse colon	15 (15.8)	21 (17.5)
Ascending colon	12 (12.6)	26 (21.7)
Cecum	8 (8.4)	6 (5%)

FICE: Fujinon intelligent color enhancement.

**Table 2:** Capillary meshwork (CM) by digital chromoendoscopy (FICE) and histopathologic findings.

	Neoplastic	Non-neoplastic
CM positive	66	1
CM negative	6	22

FICE: Fujinon intelligent color enhancement.

**Table 3:** Comparison between digital chromoendoscopy (FICE) and real-time chromoendoscopy (indigo carmine) in differentiating neoplastic from non-neoplastic lesions.

	FICE (DCE)	Indigo carmine (CE)
Accuracy % (95% CI)	92.6 (87.3–98.0)	94.2 (90.0–98.4)
Sensitivity % (95% CI)	91.7 (82.7–96.9)	96.5 (90.1–99.3)
Specificity % (95% CI)	95.7 (78.1–99.9)	88.2 (72.5–96.7)
PPV % (95% CI)	98.5 (92–100)	95.4 (88.6–98.7)
NPV % (95% CI)	78.6 (59–91.7)	90.9 (75.7–98.1)
Kappa (95% CI)	0.81 (0.68–0.95)	0.86 (0.75–0.96)

95% CI: 95% confidence interval; FICE: Fujinon intelligent color enhancement; DCE: digital chromoendoscopy; CE: real-time chromoendoscopy; PPV: positive predictive value; NPV: negative predictive value.

**Table 4:** Accuracy between superficial lesion and protruding lesion using capillary pattern (FICE) and pit pattern analysis (Indigo carmine).

	FICE (DCE)	Indigo carmine (CE)
Superficial lesion	92.7%	96.1%
Protruding lesion	92.5%	90.7%

FICE: Fujinon intelligent color enhancement; DCE: digital chromoendoscopy; CE: real-time chromoendoscopy.
